# The impact of hand dominance on haptic and visual size comparisons

**DOI:** 10.1007/s00426-026-02332-3

**Published:** 2026-07-15

**Authors:** Peter Wühr, Herbert Heuer

**Affiliations:** 1 Department of Psychology, TU Dortmund University, Emil-Figge-Strasse 50, 44227 Dortmund, Germany; 2 Leibniz Research Center for Working Environment and Human Factors (IfADo), Ardeystr. 67, 44139 Dortmund, Germany

**Keywords:** Handedness, Size comparison, Haptic modality, Visual modality, Hand strength

## Abstract

The present study investigated the impact of handedness on haptic and visual size comparisons. Moreover, we sought to determine whether strength differences between the hands are correlated with lateral differences between judged sizes. Therefore, 80 left-handed and 80 right-handed young adults (without additional constraints on individual characteristics except for those required by the experimental tasks) completed a bimanual haptic size-comparison task, a visual size-comparison task, and strength measurements of fingers, hands, and arms. In the haptic size-comparison task, we observed an impact of handedness: Both left- and right-handers underestimated the size of an object in their dominant hand relative to an object in their non-dominant hand. This hand-related bias was confined to the haptic modality; in the visual size-comparison task, we observed a small overestimation of stimulus size in the right visual hemifield, as compared to the left visual hemifield, in right-handers, but not in left-handers. The strength measurements revealed the expected superiority of the dominant effector over the non-dominant effector, but the strength asymmetries were not correlated with the bias in haptic size-comparisons. The observed impact of handedness on haptic size judgments represents a case of embodied perception in the haptic modality.

## Introduction

A major and perhaps even the primary purpose of perception is to enable bodily actions that are adapted to the environment. Thus, the stream of sensory input tends to be interpreted in terms of what our body can do with it (Bridgeman & Tseng, 2011). Indeed, effects of bodily dispositions or states on perceptual judgements are well documented, particularly for the visual modality. For example, distances are judged as longer and hills as steeper when one carries a heavy backpack (e.g., Bhalla & Proffitt, 1999; Proffitt et al., 2003). Such phenomena are referred to as „embodied perception“ (cf. Proffitt, 2006; Witt, 2011, for reviews), although their perceptual versus judgmental origin is debated (cf. Firestone & Scholl, 2016). Notably bodily variations exist even without backpacks and within each individual. A conspicuous one is the difference between the left and right hand in terms of their capabilities and the functions they serve. Here we inquire into differences between the two hands in the haptic perception of size and relate such differences to differences in the visual perception of size and to strength differences between the hands.

### Haptic perception and hand dominance

Haptic perception is the perception of the characteristics of objects and surfaces by active exploration with the fingers and hands (see, Kappers & Bergmann Tiest, 2013; Lederman & Klatzky, 2009, for reviews). Here we focus on the perception of size. For example, with the finger-span method (e.g., Kelvin & Mulik, 1958; Stevens & Stone, 1959) observers grasp an object between the thumb and the index or middle finger of their hand, and grip aperture provides a measure of object size. Size judgments with this method are fairly accurate: human observers can perceive size differences as small as 1 mm (e.g., Dietze, 1961; Durlach et al., 1989; Gaydos, 1958).

The finger-span method can be used with both hands, but the capabilities and functions of the left and right hand are strikingly different in most humans. For example, the dominant hand is usually stronger than the nondominant hand, and the two hands take different roles in bimanual action. Such differences could give rise to different haptic perceptions of object size. In fact, more than 70 years ago McPherson and Renfrew (1953) reported such differences. We shall describe this study in some detail because we sought to replicate the main finding.

McPherson and Renfrew (1953) collected comparative size judgments from 40 left-handed and 40 right-handed adults, which represents an unusually large sample for that time. Handedness was assessed on the basis of several tests (e.g., writing, drawing, polishing a table, throwing a ball, grip strength), and the results of these tests matched the self-classification of participants. Five aluminum discs of equal weight served as stimuli. A disc with a diameter of 40.0 mm was the standard (or reference) stimulus, and four discs of variable size (40.0, 41.5, 43.0, 44.5 mm) served as comparison stimuli. During the test, participants were blindfolded and sat at a table. In one half of the 32 trials, they received the standard in their left hand and a variable comparison stimulus in their right hand. In the other half of the trials, participants received the standard in their right hand and the comparison stimulus in their left hand. In half of the trials with the standard in each hand they had to judge whether the disc in their right hand was smaller, equal or larger than the disc in their left hand, and in the other half whether the disc in their left hand was smaller, equal or larger than the disc in their right hand.

For analyzing their data, McPherson and Renfrew counted the number of “smaller”, “equal”, and “larger” judgments for each difference between the two discs, which varied between − 4.5 mm and + 4.5 mm. They then used a scoring system that assigned different weights to each judgment depending on the correctness of the judgment and the difference between the to-be-judged stimuli. The smallest weight (1) was assigned to correct judgments with a large stimulus difference (the most likely response), and the largest weight (64) was assigned to an erroneous judgment with a large stimulus difference (the least likely response). Then the authors separately summed up the scores for all judgments in which the left disc was judged larger or equal to the right disc, and all judgments in which the right disc was judged larger or equal to the left disc. If the two hands of a participant produced symmetrical judgments, the two scores should be equal. A positive difference between them reflected an overestimation of the size of objects in the left hand relative to objects in the right hand, and a negative difference reflected an overestimation of the size of objects in the right hand relative to objects in the left hand.

McPherson and Renfrew (1953) computed the score differences for left- and right-handers and obtained a mean difference score of −31.7 for left-handers and a mean difference score of 59.1 for right-handers. Both scores were significantly different from zero. The negative score means that left-handers, on average, judged the discs in their left hand to be smaller than the discs in their right hand, and the positive score means that right-handers, on average, judged the discs in their right hand to be smaller than the discs in their left hand. In other words: Both groups underestimated the size of discs in their dominant hand relative to the size of discs in their non-dominant hand. A potential problem of this data analysis, however, is that it assigned very strong weights to incorrect judgments involving large differences between the stimuli. This may have amplified small effects, pushing them towards ‘significance.’

Subsequent attempts to replicate the study of McPherson and Renfrew (1953) produced mixed results. Wertheimer (1954) replicated the relative underestimation of object size in the dominant hand for right-handers (*N* = 56), but not for left-handers (*N* = 9). Other studies failed to replicate the impact of hand dominance on size judgments in right-handers (Churchill, 1971; Costello, 1961; Walker, 1972). However, a closer look at studies reporting negative results revealed that most of these studies had much smaller samples (Churchill: 10 right-handers; Costello: 18 right-handers; Walker: 32 right-handers in Experiment 1, 12 right-handers in Experiment 2) as compared to the study of McPherson and Renfrew (1953). We computed the effect sizes for left-handers (*d* = −0.371) and right-handers (*d* = 0.696) from the latter study and used them to estimate the sample sizes required for a successful replication with high power (1-β = 0.95). The result was that, at least, 24 right-handers, and, at least, 81 left-handers are required for a successful replication of McPherson and Renfrew (1953). The minimal sample size for right-handers was only achieved in the studies of Wertheimer (1954) and Walker (1972, Experiment 1), but never for left-handers. Hence, a goal of our study was to replicate the results of McPherson and Renfrew (1953) with sufficiently large samples, and without using their arbitrary scoring system.

In addition, we sought to obtain insights into the specific sources of the lateral asymmetry of haptic size perception. In principle perceptions are shaped by sensory input and expectations, in Bayesian terms: likelihoods and priors (cf. de Lange et al., 2018). Thus, in the following we discuss differences between the capabilities and functions of the left and right hand which could modulate the relevant sensory input to the two hands as well as the distribution of object sizes encountered by the hands which could modulate expectations. Before that, however, we shall briefly turn to the individual variation of hand dominance and their assessment.

### Handedness

Almost all human adults are aware of their handedness, typically defined as „the individual’s preference to use one hand predominantly for unimanual tasks and/or the ability to perform these tasks more efficiently with one hand“ (Corey et al., 2001, p. 144). The majority of them are right-handers (≈ 90%) and the minority are left-handers (≈ 10%; cf. Papadatou-Pastou, Ntolka, Schmitz, Martin, Munafò, Ocklenburg, & Paracchini, 2020). However, handedness is not a clear-cut dichotomy, but there are also people with mixed hand preferences, usually referred to as ambidextrous.

Handedness is typically assessed by means of questionnaires, with the „Edinburgh Handedness Inventory“ (EHI; Oldfield, 1971) being probably the most popular one. In the original version of the EHI, participants are asked to indicate whether they use the left or right hand for everyday activities by a + or ++ (the latter if “the preference is so strong that you would never try to use the other hand unless absolutely forced to”, Oldfield, 1971, p.112) or by a + for each hand if both hands are used. By multiplying (with 100) the ratio of the difference between the numbers of pluses for the right and left hand and the sum of the pluses, a “Laterality Quotient” is computed that varies between − 100 and + 100 and has an asymmetric U-shaped distribution: many cases with high positive values, few cases with low negative values, and even fewer cases around zero (e.g. Oldfield, 1971, Fig. 3). Since its first publication, the EHI was used in thousands of studies, but the authors often varied the original procedure (cf. Edlin et al., 2015).

Although both left-handers and right-handers prefer one hand, various differences between the hands are more pronounced for right-handers than for left-handers. Notwithstanding this difference between left-handers and right-handers, we refer to a dominant and a non-dominant hand. So, what are differences between the capabilities and functions of the hands that could underlie the difference in haptic size perception?

### Haptic size perception and differences between hands in physical and neural characteristics

An object held in a strong hand could appear smaller than an object held in a weak hand. In fact, the dominant hand is usually stronger than the non-dominant hand (cf. Bohannon, 2003; Foley et al., 2025, for reviews). Most of the studies investigated grip strength with dynamometers, which measure the strength of the so-called power grip, but the strength of fingers when performing so-called key pinch or precision grips has also been studied. Superiority of the dominant hand was reported both for hand-grip strength (e.g., Bardo et al., 2021; Peters, 1990; Triggs et al., 2000; Werle et al., 2009; Wühr et al., 2024), and for finger pinch strength (e.g., Werle et al.,, 2009; Wühr et al., 2024). Some studies showed a superiority of the dominant hand only in right-handers, but not in left-handers (e.g., Armstrong & Oldham, 1999; Crosby et al., 1994; Petersen, Petrick, & Conklin, 1989), which can often be attributed to the fact that the sample of left-handers was much smaller than that of right-handers (Werle et al., 2009). In the present experiment we tested a possible role of the strength difference between the hands for the perceived size difference by way of examining the inter-individual covariation of these two hand differences.

The hypothesis of a possible role of strength differences gains plausibility from the results of a recent study where Wühr et al. (2024) observed correlations between strength asymmetries and the size of an SSARC (“spatial-size association of response codes”) effect. This effect reflects the observation that participants respond faster by pressing a left than a right key to a small stimulus, whereas they respond faster by pressing a right than a left key to a large stimulus (e.g. Wühr & Heuer, 2024; Wühr & Seegelke, 2018). Wühr et al. (2024) hypothesized that the SSARC effect might reflect the habit of people to grasp larger objects more often with their stronger dominant hand than with their weaker non-dominant hand. Consistent with this idea, the SSARC effect (i.e., the superiority of the small-left/large-right mapping over the small-right/large-left mapping) is larger for right-handers than for left-handers, and the stronger the right hand as compared to the left hand, the larger is the SSARC effect (Wühr et al., 2024).

Although here we focus on strength differences as a possible variable related to differences of perceived size, there are other candidates such as differences in hand size. However, anthropometric studies of the size and shape of the two hands in left- and right-handers have produced only mixed results. For example, Purves et al. (1994) observed that the right hand had a larger volume than the left hand in right-handers, while the two hands did not differ in left-handers. Other studies observed larger limbs on the dominant side relative to the non-dominant side both in right- and in left-handers. For example, Peters (1990) reported that the circumference of fingers (thumb, index finger) was larger on the dominant hand than on the non-dominant hand both in left- and in right-dominant participants. Yet, the same author neither found differences in the length of the two hands, nor in the length of fingers of both hands. At present, the small number of studies and the inconsistent pattern of results prevent robust conclusions about size and shape differences between dominant and non-dominant hands.

Rather than anthropometric differences between the hands, differences in their perceived size could be crucial for the difference in haptic size perception. In fact, at least in right-handers the right hand tends to be judged larger than the left hand, a difference that is correlated with the strength difference (Linkenauger et al., 2009). Although one could expect that an object held in the phenomenally larger hand should appear smaller than the same object held in the phenomenally smaller hand, one could also expect the opposite. In fact, with experimentally induced changes of felt hand size, the size of hand-held objects was smaller when the felt size of the hand was reduced than when it was increased (Bruno & Bertamini, 2010). Thus, it seems unlikely that the apparent size difference between hands underlies the hand difference in haptic size comparisons.

Regarding neural differences between the hands, differences in the processing of somatosensory stimuli are of particular interest. For example, an underestimation of object size in the dominant hand might be related to a smaller representation or weaker electrophysiological responses to sensory stimulation in the respective somatosensory areas of the brain than in areas related to the nondominant hand. For example, Sörös et al. (1999) used Magnetencephalography (MEG) to investigate the size of somatosensory areas in the two hemispheres of left- and right-handers and observed a clear asymmetry favoring the right hand in right-handers (*N* = 7), while the pattern was more mixed in left-handers (*N* = 5). Jung et al. (2003) stimulated the left and right median nerves at the two hands and observed a stronger sensory-evoked potential (N20) over the left precentral cortex than the right one in a sample consisting mainly of right-handed participants (14 right-handers, 2 left-handers). From such lateral differences in the cortical somatosensory representations one would probably expect that the size of the object in the dominant hand would be overestimated rather than underestimated.

### Haptic and visual size perception

Lateral differences in the haptic perception of size could be specific for the haptic modality or just an instance of a generalized asymmetry apparent in other modalities as well. Therefore, we also measured asymmetries of visual size perception and their relation to asymmetries of haptic size perception. Generalized asymmetries of size perception could be a consequence of differences in various structural and functional characteristics, including perceptual ones (see Güntürkün et al., 2020, for review), between the cortical hemispheres, which are associated with handedness (Annett, 2002; Johnstone et al., 2021).

The small number of studies on asymmetries of visually perceived size of stimuli in the two visual hemifields produced mixed results (in contrast to visual asymmetries in letter identification, cf. Bryden, 1973, White, 1969). Brown (1953) observed that four out of six participants (handedness not reported) overestimated the length of a horizontal line in the right visual hemifield, relative to a line in the left visual hemifield. In contrast, Walker (1972) observed in two experiments that right-handed participants overestimated the size of an object (a block of plexiglas) presented at a left location, as compared to an object presented at a right location—a result that seems to fit the findings of McPherson and Renfrew (1953). Notably, in the study of Walker (1972) participants could freely move their eyes, so that the results cannot be straightforwardly attributed to differences between cortical hemispheres. Only a condition, in which participants fixate between the objects, would have ensured that each object is projected to visual areas in different hemispheres.

### Haptic size perception and differences in manual skills

Hand dominance is associated with differences in manual skills. These are prominent for right-handers, for example, in a pegboard test, where a row of small pegs has to be moved from one location to another (Annett et al., 1979), or for rapid finger oscillations (Heuer, 2007). Although for left-handers the differences between the hands tend to be less pronounced, they are present, for example in writing (e.g., Provins & Magliaro, 1993), in pegboard tasks (e.g., Bryden & Roy, 2005; Schmidt, Oliveira, Rocha, & Abreu-Villaça, 2000; Triggs et al., 2000), and finger tapping (e.g., Peters & Servos, 1989; Triggs et al., 2000). However, not all tasks reveal a superiority of the preferred hand. For example, in right-handers unexpected inertial loads were more efficiently compensated by the non-dominant than by the dominant arm (Bagesteiro & Sainburg, 2003).

The distinction between dominant and non-dominant hand loses its “better versus worse” characteristic when one examines bimanual actions in which the hands cooperate (Peters, 1981; Guiard, 1987). This cooperation is characterized by a specific functional specialization: the non-dominant hand typically holds or stabilizes objects so that the dominant hand can manipulate them or parts of them. An everyday example is opening a bottle with a screw cap, where the non-dominant hand typically holds the bottle and the dominant hand manipulates the cap; another example is dealing cards. In this type of bimanual cooperation, the two hands operate on different spatio-temporal scales. Guiard (1987) describes the difference as a micrometric and a macrometric specialization. The different temporal scales are also apparent in rhythm production (Ibbotson & Morton, 1981): it is easy to tap a steady beat with the non-dominant hand together with a rhythm of the dominant hand, but performance with the opposite hand assignment is (almost) impossible. The same is true for tapping in pace with a metronome with one hand, while tapping as rapidly as possible with the other hand, particularly for right-handers (Peters, 1981).

The haptic size-comparison task of McPherson and Renfrew (1953) is a bimanual task. Therefore, there could be the expectation of a larger object in the non-dominant hand than in the dominant one, or – to avoid the possible connotation of conscious awareness – a corresponding prior. Such expectations or priors are shaped by the “statistics of everyday-life” and could be resistant to experimental manipulations, though possibly modifiable across several days as, for example, the relation between object size and expected weight (e.g. Flanagan et al., 2008). Note that size priors for bimanual tasks can be different from those for unimanual tasks. Specifically, as described above, for unimanual tasks the stronger dominant hand might more frequently deal with larger objects than the non-dominant hand rather than with smaller objects as in bimanual tasks.

### The present study

The main purpose of the present study is to replicate a fundamental empirical phenomenon of human perception and to identify its sources in order to contribute to a theoretical account of it. Our inductive research strategy comprised three steps. First, we attempted to replicate the results reported by McPherson and Renfrew (1953), but without using their arbitrary scoring system and with more statistical power. Second, concerning possible sources of the bias, we assessed strength differences between the two hands as a possible source for the expected impact of hand dominance on haptic size judgments. Therefore, we assessed correlations between the size of strength differences between the hands, and the size of the hand-related bias in haptic size comparisons. Third, in a more exploratory manner, we tested the impact of handedness on visual size comparisons with stimuli presented in the left and right visual hemifields. This was done to explore the possibility of some broad perceptual asymmetry (related to handedness) of size perception independent of the sensory modality.

## Methods

### Openness and transparency

We have preregistered this study in April 2024 on the OSF platform [https://osf.io/zc7fb]. The local Ethics Committee at TU Dortmund University approved the experimental protocol of the study (GEKTUDO_2024-14). We report how we determined our sample size, data exclusion, all manipulations, and all measures in the study. The vocal responses in the haptic size-comparison task were noted on paper sheets by research assistants, summed up, and entered into Excel tables. The table containing the data from the haptic size-comparison task and the raw data files from the visual size-comparison task have been published on the OSF platform (https://doi.org/10.17605/OSF.IO/XVE54).

### Participants

We computed effect sizes for the bias effects in left- and right-handers, as reported in McPherson and Renfrew (1953), and used these values for a power analysis with G*Power (Faul et al., 2007). This analysis revealed that sample sizes of 24 right-handers and 81 left-handers, respectively, are required for replicating the original findings with high power (1 - β = 0.95) at the usual 0.05 alpha error probability. Therefore, we decided to test 80 left- and 80 right-handers.

Participants were recruited through advertisements on internet platforms for students of TU Dortmund University, and by posters on the university campus. Participants gave their informed consent prior to participation and received either course credit or a payment of 10 Euro. All participants had intact upper limbs, and reported fully intact hand function, as well as normal or corrected-to-normal vision. Before testing, we assigned participants to handedness groups based on self-classification. After testing, we compared self-classification to the results of a handedness questionnaire, which was a modified version of the Edinburgh Handedness Inventory (EHI) suggested by Williams (2020). Participants indicated on a five-point rating scale how often they performed each of 8 activities with the left or right hand. The activities were writing, throwing, using scissors, using a tooth-brush, using a knife, using a spoon, lighting a match, and operating a computer mouse. The answers were coded as −2 (always left), −1 (mostly left), 0 (sometimes left, sometimes right), 1 (mostly right), and 2 (always right) and summed up. The sum was used to compute a handedness score using the formula *HS* = (sum/16)*100. The score ranges from − 100 to + 100. Following Oldfield (1971), participants with a positive score were classified as right-handers, whereas participants with a negative score were classified as left-handers.

Eighty participants classified themselves as right-handers. The results of the handedness questionnaire confirmed the self-classifications: The mean handedness score was 86.41 (median: 87.50, SD: 15.30, range: 37.50 to 100). All right-handers used their right hand for writing. The sample of right-handers consisted of 70 females and 10 males, with an average age of 22.86 years (*SD* = 4.73).

Eighty other participants classified themselves as left-handers. However, the results of the handedness questionnaire confirmed these self-classifications for 76 participants only, whereas four participants had positive EHI scores ranging from 6.25 to 62.50. These participants were replaced by four new participants. The final sample included 80 left-handers with an average handedness score of −58.44 (median: −62.5, SD: 25.26, range: −6.25 to −100). The huge majority of left-handers (i.e. 79 participants) always used their left hand for writing. However, the majority of left-handers (i.e. 52) used their right hand for operating the computer mouse at least often, which reduced the absolute mean handedness score for the left handers compared to the right handers. The sample of left-handers consisted of 54 females and 26 males, with an average age of 24.03 years (*SD* = 5.78).

### Apparatus and stimuli

**Fig. 1:**
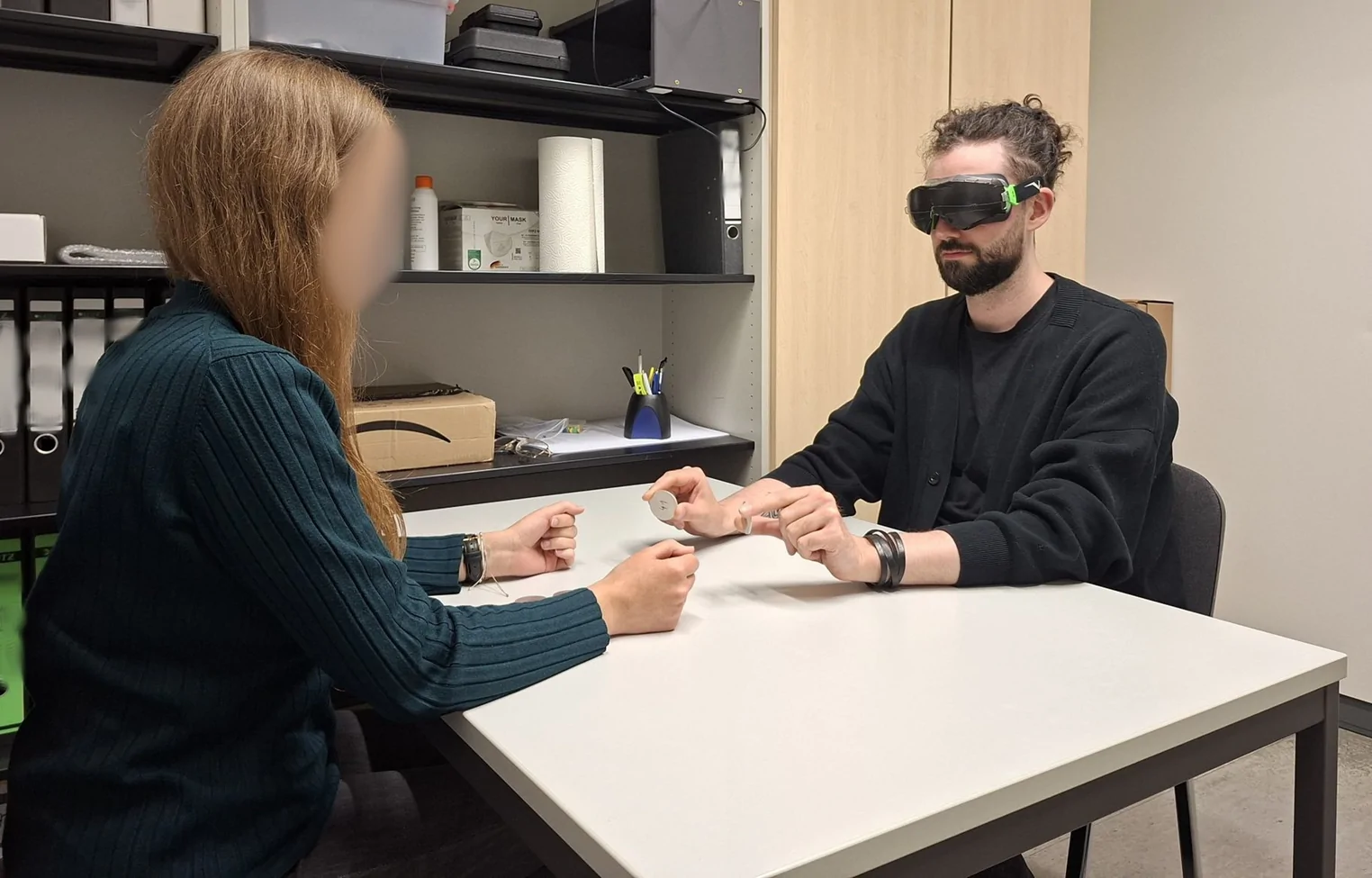
The picture shows the experimenter (to the left) and a blind-folded participant (to the right) during the haptic size-comparison task

For the haptic size-comparison task, we used ten custom-made aluminum discs. The discs had diameters of 38, 39, 40, 41, and 42 mm, with two equal discs for each diameter. All discs were 2 mm thick. The weights of the five discs with ascending size were 5.5 g, 6.0 g, 6.5 g, 7.0 g, and 7.5 g. We did not attempt to equalize the weights of the discs for several reasons. First, McPherson and Renfrew (1953) used discs of equal weight, showing that size differences between their discs were the main source of the observed bias in haptic judgments. They should also be a source in a replication of this bias, even when weight differences do exist. Second, these mass differences were rather small and very likely below the just-noticeable difference (JND), especially considering that JNDs can also increase when participants are blindfolded (e.g., Wolf & Drewing, 2020). Third, attempts to equalize weight (e.g., by attaching adhesive tape to the discs) might have caused other features of the disc (e.g., thickness) to correlate with size. Finally, several studies failed to find evidence for an underestimation of weight in the dominant hand in right- and left-handed participants (Brodie, 1988; Saslow & Watkins, 1978; Shen, 1935). For blindfolding our participants, we used a pair of protective goggles that fully covered their visual field. The transparent parts of the goggles were covered with black adhesive tape (cf. Figure 1).

For the visual size-comparison task, participants sat in front of a 24-inch monitor with a viewing distance of approximately 50 cm in a normally lighted room. We used the software E-Prime 3.0 (Psychology Software Tools; Sharpsburg, PA, USA) to control the presentation of stimuli and register responses (i.e., key pressed, reaction time [RT]). A plus sign (Courier font, size 18 pt) served as a fixation point at the beginning of each trial. The stimuli were filled white circles with variable diameters of 37, 38, 39, 40, 41, 42, and 43 mm. respectively. Stimuli were presented in pairs, with one stimulus to the left and the other stimulus to the right of fixation. The distance between the center of each stimulus and fixation was 15 cm; hence, the distance between two stimuli—as measured from center to center—was 30 cm. When viewed from a distance of 50 cm, the distance between two stimuli was approximately 33.4° of visual angle. All stimuli were presented in white on a black background.

We used a hydraulic pinch gauge by Saehan (SH5005, Saehan corporation, South Korea) to measure participants’ finger pinch strength and a hand dynamometer by Saehan (SH5001, Saehan corporation, South Korea) to measure participants’ hand grip strength. For the measurement of arm strength, we employed a digital hanging scale by PCE instruments (PCE-HS 150 N, PCE Deutschland GmbH, Germany). We attached a solid exercise handle to the top end of the scale which was held by the participants. An inelastic but adjustable strap attached to the bottom end of the scale ended in a loop which was fixed to the floor when participants stepped on it. Strength was measured in kilograms (kg).

### Procedure

Each participant performed five different tasks. Three of these tasks were strength measurements, where we measured (a) pinch strength of both hands, (b) hand-grip strength of both hands, and (c) lifting strength of both arms. Each strength measurement was performed twice for both the left and right hand/arm. One series of six measurements (3 effectors × 2 sides) was made at the beginning of the experiment, and a second series of six measurements was made at the end of the experiment. The haptic and the visual size-comparison tasks were performed between the strength-measurements. Overall, we included eight different orders of tasks that were counterbalanced across participants (see Table 1). Each participant performed the tasks in one of these orders. Hence, there were 10 participants with each possible order in both handedness groups.

**Table 1:** Possible orders of five tasks, including repetitions of the strength-measurement tasks

Order	Task							
	Arm 1	Hand 1	Finger 1	Size 1	Size 2	Finger 2	Hand 2	Arm 2
1	L-R	L-R	L-R	Hap 1a	Vis 1	R-L	R-L	R-L
2	L-R	L-R	L-R	Hap 2a	Vis 2	R-L	R-L	R-L
3	L-R	L-R	L-R	Hap 1b	Vis 1	R-L	R-L	R-L
4	L-R	L-R	L-R	Hap 2b	Vis 2	R-L	R-L	R-L
5	R-L	R-L	R-L	Vis 1	Hap 1a	L-R	L-R	L-R
6	R-L	R-L	R-L	Vis 2	Hap 2a	L-R	L-R	L-R
7	R-L	R-L	R-L	Vis 1	Hap 1b	L-R	L-R	L-R
8	R-L	R-L	R-L	Vis 2	Hap 2b	L-R	L-R	L-R

Note. L-R = left-right; R-L = right-left; Hap 1a = haptic size-comparison task with response to larger stimulus and trial order a; Hap 1b = haptic size-comparison task with response to larger stimulus, and trial order b; Hap 2a = haptic size-comparison task with response to smaller stimulus and trial order a; Hap 2b = haptic size-comparison task with response to smaller stimulus and trial order b; Vis 1 = visual size-comparison task with response to larger stimulus; Vis 2 = visual size-comparison task with response to smaller stimulus

#### Haptic size-comparison task

For the *haptic* size-comparison task, the participant and the experimenter sat vis-à-vis on opposite sides of a table. The participant was blindfolded by opaque goggles. The diameter was written on each disc for the experimenter. In each trial, the experimenter took a pair of discs, as indicated on a paper list, and gave them to the participant, one disc in each hand. The participants were told to hold the discs between the tips of the thumb and the index finger of each hand, with hands in a palm-down position (cf. Figure 1), and they were allowed to roll the discs between the two fingers. The participants’ task was to haptically compare the size of the two discs and to indicate the side of the larger or smaller disc. In particular, half of the participants always indicated the side of the larger disc (conditions “Hap 1a” and “Hap 1b” in Table 1), whereas the other half of the participants always indicated the side of the smaller disc (conditions “Hap 2a” and “Hap 2b” in Table 1). The experimenter recorded each response in a list. There were two orders (a and b) for presenting stimulus pairs to the participants. Order (a) was randomly determined, and order (b) was the reverse of (a). The counterbalancing of trial order is shown in Table 1.

The haptic size-comparison task consisted of 70 trials, 10 trials with each of 7 right-minus-left differences between the diameters of the discs held in both hands: −3, −2, −1, 0, + 1, +2, and + 3 mm. Different from the classic Method of Constant Stimuli (e.g. Baird & Noma, 1978, pp. 37–39), these differences were not defined as differences between a constant standard stimulus and a variable comparison stimulus, but by differences between various combinations of the different sizes of the discs as listed in Table 2. (Note that the sum of the frequencies along each diagonal, where the size difference is constant, is 10.) With this modification of the standard method we avoided the presentation of one and the same disc in each trial, but in turn could not estimate points of subjective equality for a specific size of a standard disc, but only for a small range of sizes. This was appropriate for the purpose of our study because we expected lateral asymmetries of size perception across a range of sizes. There were two different pseudo-random orders of pairs of discs for this task (called [a] and [b] in Table 1) which were counterbalanced across participants.

**Table 2:** Frequency of pairings of disc sizes in the haptic size-comparison task. Frequencies in the main diagonal and those parallel to it sum up to 10, which is the number of repetitions of a certain left-right difference

		Size of Disc in Right Hand				
		38	39	40	41	42
Size of Disc in Left Hand	38	2	3	4	5	0
	39	3	2	2	2	5
	40	4	2	2	2	4
	41	5	2	2	2	3
	42	0	5	4	3	2

Note: Different combinations of two sizes were shown with different frequencies in order to match the frequencies of size differences. The 38-mm disk was never compared with the 42-mm disk

#### Visual size-comparison task

For the *visual* size-comparison task, the participant sat at a table in front of a computer screen. The computer first presented written instructions and showed an example for a visual display on the screen. Then there was a short practice block with 9 trials, and two test blocks with 66 trials each. In each trial, two circles of the same or of different size were presented on the screen, one circle to the left and the other circle to the right of fixation. The 132 trials of the two test blocks consisted of 12 trials for each of 11 right-left differences of −5, −4, −3, −2, −1, 0, + 1, +2, + 3, +4, and + 5 mm between the diameters of the two circles. Because the duration of a trial was shorter in the visual task, as compared to the haptic task, we used more stimulus sizes in the visual task, allowing us to explore a larger range of sizes. For this task we deviated from the classic Method of Constant Stimuli in the same way as for the haptic size-comparison task. The frequencies of the combinations of specific stimulus sizes are shown in Table 3. These combinations were presented in random order.

**Table 3:** Frequency of pairings of disc sizes in the visual size-comparison task. Frequencies in the main diagonal and those parallel to it sum up to 12, which is the number of repetitions of a certain left-right difference

		Size of Circle in Right Screen Location						
		37	38	39	40	41	42	43
Size of Circle in Left Screen Location	37	1	2	2	3	4	6	0
	38	2	2	2	2	3	4	6
	39	3	2	2	2	3	3	4
	40	3	2	2	2	2	2	3
	41	4	3	3	2	2	2	3
	42	6	4	3	2	2	2	2
	43	0	6	4	3	2	2	1

Note: Different combinations of two sizes were shown with different frequencies in order to match the frequencies of size differences

Each trial of the visual size-comparison task started with a blank screen for 500 ms, followed by a fixation point at screen center for another 500 ms. Then, two circles were shown to the left and right of fixation, respectively, for 200 ms. Participants had been instructed to maintain central fixation. When the circles had disappeared, a question appeared on the screen. For half of the participants, the question was “Which circle was bigger? Left or right?” (condition “Vis 1” in Table 1). For the other half of the participants, the question was “Which circle was smaller? Left or right?” (condition “Vis 2” in Table 1). The question remained until the participant had pressed a key, but only for a maximal duration of 1,800 ms. For responding, participants pressed the left Control key (at the lower-left edge of the keyboard) with the left index finger or the right Enter key (at the lower-right edge of the keyboard) with the right index finger. Registration of responses started with the onset of the two discs and lasted for a maximal duration of 2,000 ms. After a response, or when 2,000 ms had elapsed without a response, a blank screen was again shown for 500 ms. There was no feedback with regard to speed or accuracy of the response.

#### Strength measurements

To measure finger (grip) strength, we used the key pinch (thumb pulp pressed to lateral aspect of middle phalanx of the index finger) and followed the Clinical Assessment Recommendations provided by the American Society of Hand Therapists (MacDermid et al., 2015). To measure hand (grip) strength, participants were in a standing position and the experimenter followed the procedure described by Mathiowetz (1990). To ensure standardization, the hand dynamometer was set to the second handle position for all participants since sufficiently accurate measurements of grip strength can be obtained with this position for all subjects (Trampisch et al., 2012). To measure arm (lifting) strength, we followed a procedure described and validated by Axelsson and Kärrholm (2018) to test participants’ lifting strength with a self-constructed hanging scale dynamometer. Participants fixed the hanging scale to the floor by standing on a strap attached to the bottom of the scale, while holding a handle attached to the top of the scale in their hand. The length of the strap was adjusted to participants height to have the forearm parallel to the floor. We measured participants’ arm strength in a pronated (palm-down) position resembling lifting movements in everyday life. In all measurements, participants were instructed to apply their maximum force for at least one second.

### Data analysis

We planned to perform five sets of analyses of the data. The goal of the first analysis was to address the impact of participants’ handedness on size comparisons in the haptic size-comparison task. The main dependent variable for this analysis was the point of subjective equality (PSE) of the two discs for each participant. The PSE was determined as the mean of a psychometric function, specifically a cumulative Gaussian, fitted to the proportions of judgments at each size difference according to which the disc held in the right hand was the larger one (“right larger” for short; in practice these were the proportions of choices of the right side when the side of the larger disc had to be indicated, but the proportion of choices of the left side when the side of the smaller disc had to be indicated). Psychometric functions were fitted using the software “psignifit 4”, which provides Bayesian parameter estimates (Schütt et al., 2016). The relative underestimation of felt object size by the dominant hand relative to felt object size by the non-dominant hand should lead to a larger PSE for right-handers than for left-handers, perhaps even to a positive PSE for right-handers (larger disc in the right than in the left hand for 50% “right larger” judgments) and a negative one for left-handers.

The goal of the second analysis was to investigate the impact of participants’ handedness on visual size comparisons. The main dependent variable for this analysis again was the PSE as estimated from a psychometric function fitted to the proportions of “right larger” judgments for each participant.

The goal of the third analysis was to address the impact of handedness (left, right) as a between-subjects variable, and effector side (left, right) as a within-subjects variable, on the strength of three different effectors (i.e., finger, hand, arm). Therefore, we planned to conduct a two-factorial 2 (Handedness) × 2 (Effector Side) ANOVA for each effector, with strength (averaged across two measurements) as the dependent variable.

The goal of the fourth analysis was to explore the relationship between possible asymmetries in manual and visual size comparisons. Therefore, we planned to compute correlations between the PSEs in the two tasks.

Finally, the goal of the fifth analysis was to explore relationships between strength-differences between the two hands and asymmetries in haptic and visual size comparisons. Therefore, we planned to compute the strength difference between each pair of left and right effectors, and then to determine the correlation between the strength difference and the PSE in the haptic and the visual size-comparison task, respectively.

Before conducting pairwise comparisons, we always checked if the distribution of the dependent variable deviated significantly from a normal distribution or not. If we observed a significant deviation, we used a nonparametric test (e.g., Mann-Whitney U test) for comparison.

## Results

### Haptic size comparisons

**Fig. 2:**
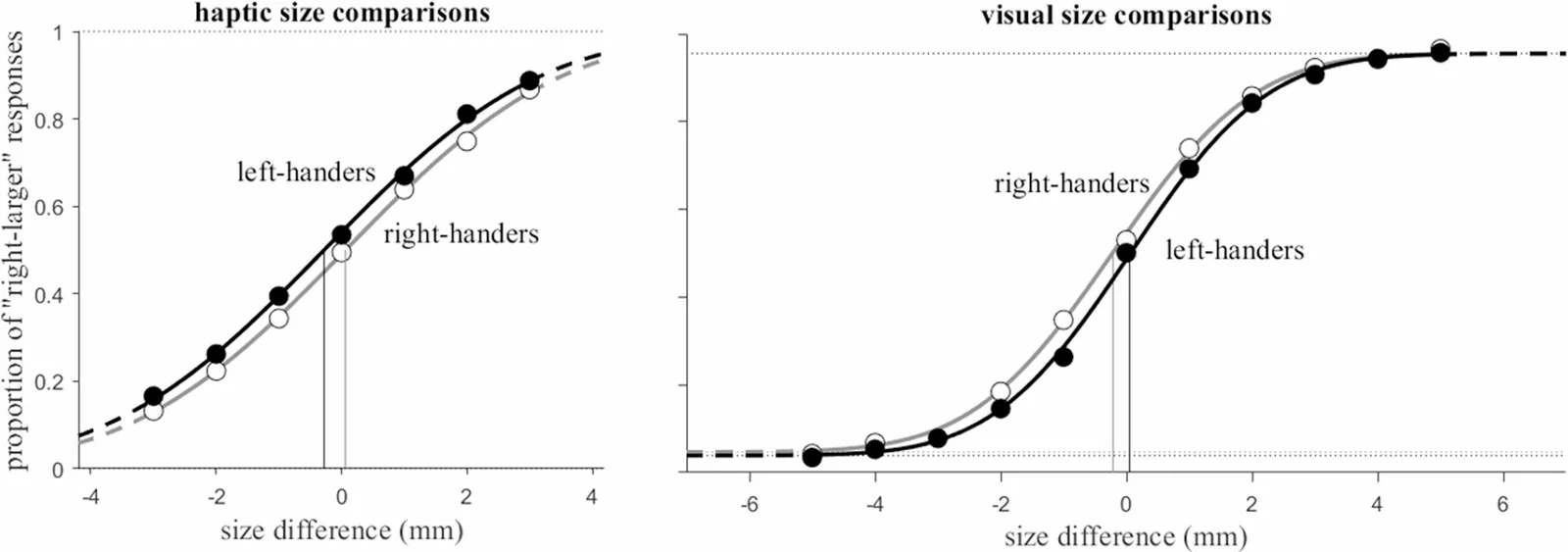
Psychometric functions for pooled data of left-handers (filled circles) and right-handers (open circles) and both haptic and visual size comparisons. Size differences are for right discs/circles minus left discs/circles, that is, the right disc/circle is larger for positive differences and the left disc/circle for negative differences. Vertical lines mark the PSEs of both handedness groups

We first analyzed the impact of participants’ handedness on haptic size comparisons. For illustrative purposes we also fitted psychometric functions to the *pooled* data of right-handers and left-handers. These are shown in Fig. 2**(left panel)**. For right-handers the psychometric function was shifted to the right as compared to left-handers, and the PSE was slightly in the range of positive right-minus-left size differences whereas for left-handers it was slightly in the range of negative differences.

For the statistical analysis we used PSEs derived from *individually* fitted psychometric functions. A one-tailed *t* test^1^, with Handedness as an independent variable, revealed that the PSE of the left-handers was significantly lower (*M* = − 0.298, *SD* = 1.018) than the PSE of the right-handers (*M* = 0.112, *SD* = 1.084), *t*(158) = 2.466, *p* =.007, *d* = 0.390. Further comparisons showed that the PSE of the left-handers was significantly smaller than zero, *t*(79) = 2.617, *p* =.011, *d* = 0.293. Thus, to appear of equal size, for left-handers the disc in the left hand had to be larger than the disc in the right hand, that is, the size of the left-hand disc was underestimated relative to the size of the right-hand disc. However, the PSE of the right-handers did not differ from zero when all participants were included in the analysis, *t*(79) = 0.925, *p* =.358, *d* = 0.103, but when four outliers—as identified by the Tukey criterion^2^—were excluded, the PSE of the right-handers (*M* = 0.192, *SD* = 0.866) was significantly larger than zero, *t*(75) = 1.933, *p* =.025, *d* = 0.222. According to this difference, right-handers had to hold a larger disc in the right hand than in the left hand for the discs to appear of equal size, that is, the size of the right-hand disc was relatively underestimated.

### Visual size comparisons

In the second analysis, we investigated the impact of participants’ handedness on visual size comparisons. The psychometric functions for the pooled data of right-handers and left-handers in this task are also shown in Fig. 2**(right panel)**. Here for right-handers the psychometric function was shifted to the left as compared to left-handers.

In the statistical analysis of individually determined PSEs, a nonparametric *U* test with Handedness as an independent variable revealed that the difference between the PSEs of left-handers (*M* = 0.051, *SD* = 1.139) and right-handers (*M* = −0.186, *SD* = 1.152) was not significantly different when all participants were included in the analysis, *U* = 2,655.50, *p* =.063, *r*_bis_ = 0.170^3^. Only when eight outliers (four left-handers, four right-handers as identified by the Tukey criterion) were excluded, the PSE of the left-handers (*M* = 0.057, *SD* = 0.682) was significantly *larger* than the PSE of the right-handers (*M* = −0.202, *SD* = 0.752), *U* = 2,345.50, *p* =.046, *r*_bis_ = 0.188. Further comparisons showed that the PSE of right-handers was significantly smaller than zero according to a Wilcoxon signed-rank test both for the full sample, *W* = 1,206, *p* =.047, *r*_bis_ = 0.256, and when outliers had been removed, *W* = 1,051, *p* =.033, *r*_bis_ = 0.282 (two-tailed)^4^. Thus, for right-handers the circle on the right side of the monitor had to be smaller than the circle on the left side to appear as being of equal size, that is, the size of the circle on the right side was relatively overestimated. In contrast, the PSE of left-handers neither differed from zero for the full sample, *W* = 1,746, *p* =.547, *r*_bis_ = 0.078, nor when outliers had been removed, *W* = 1,589, *p* =.516, *r*_bis_ = 0.086. Thus, left-handers perceived circles of identical size as being identical without a systematic over- or underestimation of circles presented in the one or the other lateral hemifield.

### Strength measurements

In our third analysis, we addressed the impact of Handedness (left, right) as a between-subjects variable, and Effector Side (left, right) as a within-subjects variable, on the strength of three different effectors (i.e., finger, hand, arm). For this analysis the results of the two measurements before and after the size-comparison tasks were averaged for each combination of effector and effector side. Averaging seems appropriate because the correlations between the two measurements were very high, ranging from 0.817 (for the right arm) to 0.937 (for the left hand). Combining the two measurements of the same effector into a single measure for later analysis was planned from the outset.

**Fig. 3:**
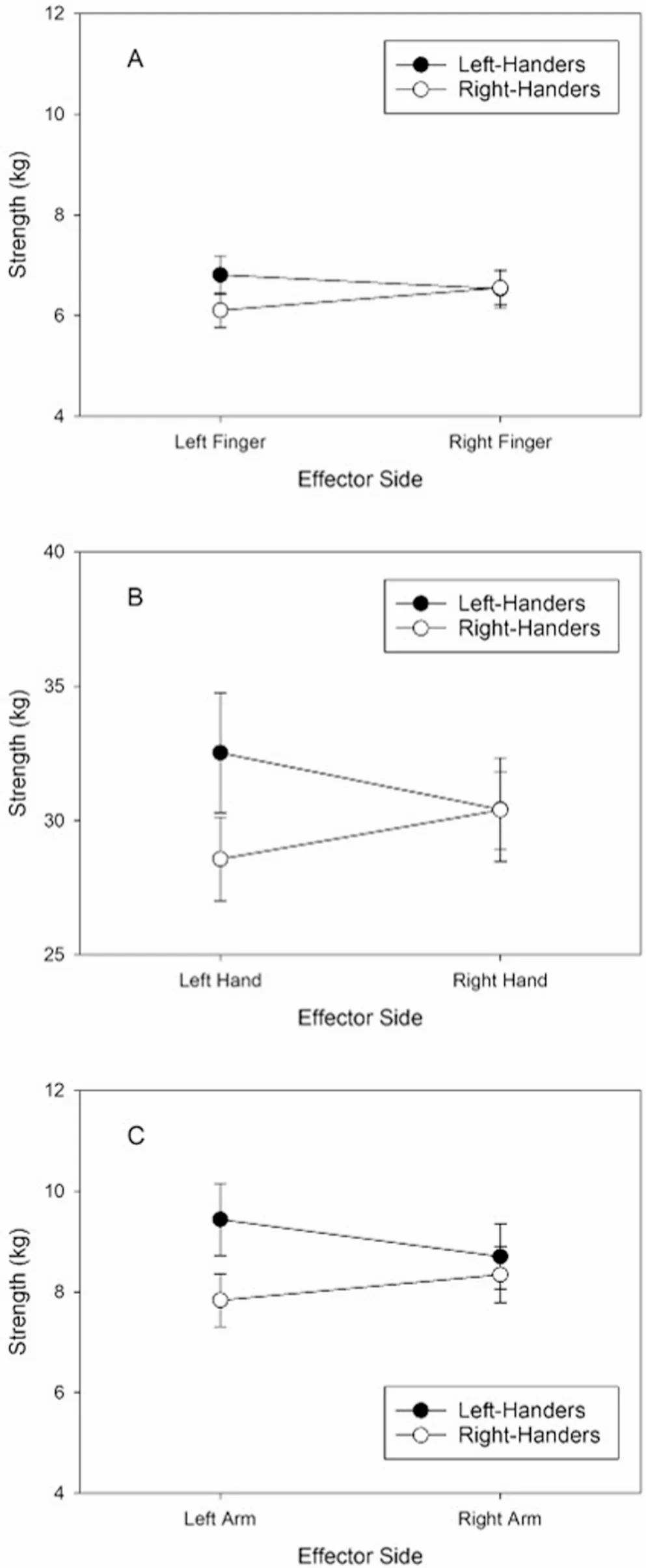
Mean strength measurements of left-hand and right-hand fingers (Panel **A**), left and right hands (Panel **B**), and left and right arms (Panel **C**), measured in left-handers (filled circles) and right-handers (open circles). Error bars reflect 95%-confidence intervals for between-subjects designs

A 2 × 2 ANOVA, with maximal strength of fingers as the dependent variable, revealed a significant two-way interaction of Effector Side and Handedness, *F*(1, 158) = 26.514, *MSE* = 0.396, *p* <.001, $\:{\eta\:}_{p}^{2}$ = 0.144. The interaction reflected the finding of higher grip strength for the dominant than for the non-dominant hand for both handedness groups (cf. Figure 3, **panel A**). Planned comparisons showed that the left fingers were stronger than the right fingers in left-handers, *t*(79) = 2.681, *p* =.005, *d* = 0.300, whereas the right fingers were stronger than the left fingers in right-handers, *t*(79) = −4.692, *p* <.001, *d* = −0.525. The main effects of Effector Side and Handedness were not significant, both *F*(1, 158) < 1.90, both *p* >.170.

A 2 × 2 ANOVA, with maximal strength of hands as the dependent variable, revealed a significant two-way interaction of Effector Side and Handedness, *F*(1, 158) = 54.078, *MSE* = 5.752, *p* <.001, $\:{\eta\:}_{p}^{2}$ = 0.255. The interaction reflected the finding of higher grasping strength for the dominant than for the non-dominant hand for both handedness groups (cf. Figure 3, **panel B**). Planned comparisons showed that the left hand was stronger than the right hand in left-handers, *t*(79) = 5.060, *p* <.001, *d* = 0.566, whereas the right hand was stronger than the left hand in right-handers, *t*(79) = −5.447, *p* <.001, *d* = −0.609. The main effects of Effector Side, and Handedness, were not significant, both *F*(1, 158) < 2.40, both *p* >.120.

A 2 × 2 ANOVA, with maximal strength of arms as the dependent variable, revealed a significant two-way interaction of Effector Side and Handedness, *F*(1, 158) = 59.738, *MSE* = 0.519, *p* <.001, $\:{\eta\:}_{p}^{2}$ = 0.274. The interaction reflected the finding of higher strength for the dominant than for the non-dominant arm for both handedness groups (cf. Figure 3, **panel C**). Planned comparisons showed that the left arm was stronger than the right arm in left-handers, *t*(79) = 6.345, *p* <.001, *d* = 0.709, whereas the right arm was stronger than the left arm in right-handers, *t*(79) = −4.554, *p* <.001, *d* = −0.509. Moreover, a significant main effect of Handedness reflected larger arm strength in left-handers than in right-handers, *F*(1, 158) = 5.052, *MSE* = 15.234, *p* =.026, $\:{\eta\:}_{p}^{2}$ = 0.031. This group difference, however, most likely reflects a sex difference because there were more men in the group of left-handers than in the group of right-handers. In fact, an exploratory analysis revealed that arm strength was higher in men than in women, *U* = 517.00, *p* <.001, *r*_bis_ = 0.768, for the left arm, *U* = 735.00, *p* <.001, *r*_bis_ = 0.670, for the right arm. The main effect of Effector Side was not significant, *F*(1, 158) = 1.984, *MSE* = 0.519, *p* =.161, $\:{\eta\:}_{p}^{2}$ = 0.274.

### Relationship between manual and visual size comparisons

In our fourth analysis, we addressed the relationship between the biases in haptic and visual size comparisons. Therefore, we computed the Pearson correlation between the PSEs in the two tasks. It turned out to be only small and not significantly different from zero, *r* =.044, *p* =.583. Small, and nonsignificant, results were also obtained when correlations were computed separately for the group of left-handers, *r* =.189, *p* =.093, and the group of right-handers, *r* = −.050, *p* =.659.

### Relationship between strength differences and size-comparison judgments

Finally, we investigated relationships between strength-differences between the two hands and biases in manual and visual size comparisons. Therefore, we first computed the difference in strength between the right and left effector for each pair of effectors (fingers, hands, and arms) and participant. Then, we computed Pearson correlation coefficients between each strength difference and the haptic PSE as well as the visual PSE. The results are shown in Table 4.

**Table 4:** Pearson correlation coefficients for strength differences between right and left effector pairs, and the PSEs in the manual and the visual size-comparison task

	Fingers	Hands	Arms
	(right-left)	(right-left)	(right-left)
Hands (right-left)	**.534**		
Arms (right-left)	.**416**	.**513**	
PSE (haptic)	− 0.073 [−0.006]	0.000 [0.070]	0.043 [0.122]
PSE (visual)	− 0.059 [−0.060]	− 0.136 [−0.157]	**− 0.167** [**−0.185**]

Note. Significant correlations (*p* <.050) are printed in bold. Numbers in brackets show correlation coefficients after participants with outlier PSE values have been excluded

The planned analyses revealed two results. Firstly, the strength differences between all effector pairs showed positive correlations, all *r* >.41, all *p* <.001. Secondly, the strength differences between right and left effectors were not significantly correlated with the PSE in the haptic size-discrimination task, all *r* <.080, all *p* >.350. This also held when four participants with outliers in their haptic PSEs were excluded, all *r* <.130, all *p* >.150. In an exploratory analysis, we determined the correlations between strength differences and the PSE in the visual task. This analysis showed a small, but significant negative correlation between the strength difference for arms and the visual PSE: The stronger the right arm relative to the left arm, the larger the right visual stimulus had to be relative to the left stimulus in order to be judged equal in size. In contrast, the correlations between strength differences for fingers and hands, respectively, and the visual PSE were not significant. This pattern was independent of whether eight participants with outliers in their visual PSEs were excluded or not.

## Discussion

In the haptic size-comparison task we observed a negative PSE for left-handed participants and a positive PSE for right-handed participants, that is, a lateral asymmetry of haptic size perception that depends on handedness: Left-handed participants underestimate the size of the disc in their left hand relative to that in their right hand, so that the disc in their left hand has to be larger than the disc in their right hand for the two discs to be judged as equal in size. For right-handers, in contrast, the disc in the right hand has to be larger than the disc in the left hand for the two discs to be judged as equal in size, that is, the size of the disc in their right hand is underestimated relative to the size of the disc in their left hand.

Our results replicate the findings of McPherson and Renfrew (1953) in spite of some procedural differences. For example, our participants judged which hand held the larger disc or which hand held the smaller disc, answering “left” or “right”, whereas the participants of McPherson and Renfrew (1953) judged whether the left hand held the larger, equal, or smaller disc or whether the right hand held the larger, equal, or smaller disc, answering “larger”, “equal”, or “smaller”. These authors were the first to show that both left-handers and right-handers underestimate the size of a disc in their dominant hand relative to the size of another disc in their non-dominant hand, but two facts raised doubts about the reliability of their findings. First, they used an unusual scoring system for analyzing their results, which assigned very strong weights to incorrect judgments involving large differences between the stimuli. This may have amplified small effects, pushing them towards ‘significance.’ Second, subsequent studies (e.g., Churchill, 1971; Costello, 1961; Walker, 1972) replicated the original findings only partially or not at all. However, the small samples of these replication studies imply insufficient statistical power: our own power analyses revealed that a sample of 80 left-handers is required for replicating the results of McPherson and Renfrew (1953) in left-handers—a sample size that was never achieved in previous replication studies. Here we show that the results of McPherson and Renfrew (1953) can be replicated without using their specific scoring system, provided the sample is sufficiently large and thus statistical power adequate for the small lateral asymmetries of the haptic perception of size.

In the following we discuss possible origins of the lateral asymmetries of haptic size perception, first the hypothesis that strength asymmetries between the upper extremities play a role, second the hypothesis that the lateral asymmetry of haptic size perception is an instance of universal lateral asymmetries in different sensory modalities. As we found no support for these two hypotheses, we subsequently turn to other possible origins. Finally, we discuss the generality of the present findings.

### Impact of strength differences on haptic size comparisons

The dominant hand is stronger than the non-dominant hand. This is an empirically well supported asymmetry both for right-handers and left-handers (cf. Bohannon, 2003; Foley et al., 2025, for reviews). The superiority of the dominant limb cannot only be observed in the grip strength of hands (e.g., Peters, 1990), but also in the strength of fingers and arms (e.g., Wühr et al., 2024). We hypothesized that objects in the stronger dominant hand could be perceived as smaller than objects in the weaker non-dominant hand, e.g. for the following reason. First, the object in the dominant hand could appear lighter than the object in the non-dominant hand, as suggested by Shen (1936, p.65): „the preferred hand, being capable of exerting greater force, should under-estimate a heavier (than the one lifted by the other hand) weight and regard it as equal to the one lifted by the non-preferred hand.“ Second, the size and the weight of objects are typically correlated. Therefore, the apparently lighter weight held in the dominant hand is likely perceived as smaller than the apparently heavier weight in the non-dominant hand (cf. Wühr et al., 2024, for a similar argument). However, testing both right- and left-handed participants, several studies failed to find direct evidence for an underestimation of a weight in the dominant hand relative to a weight in the non-dominant hand (e.g., Brodie, 1988; Saslow & Watkins, 1978; Shen, 1935). Thus, the above reasoning is plausible, but not necessarily correct.

Here we observed the expected superiority in strength of the dominant limb in three different tasks: Both for left-handers and for right-handers, the finger (when performing a key pinch), the hand (when performing a power grip), and the arm (when lifting a weight) were stronger on the dominant than on the non-dominant side, replicating similar findings by Wühr et al. (2024). However, in spite of the group difference between left-handers and right-handers, none of the strength differences (finger, hand, arm) correlated significantly with the PSE of haptic size comparisons. Hence, these results do not support the hypothesis that strength differences between dominant and non-dominant upper extremities are crucial for the asymmetry of bimanual haptic size comparisons. However, we cannot exclude the possibility that strength differences between dominant and non-dominant hands could be more impactful if the mass of the to-be-compared objects was greater than in our study.

### Impact of handedness on visual size comparisons

To test whether the asymmetry of haptic size perception is a modality-specific phenomenon or an instance of a universal lateral asymmetry, possibly related to differences between the cortical hemispheres, we tested left-handers and right-handers in a visual size-comparison task where two stimuli were shown simultaneously, one in each visual hemifield. In this task, we observed a small relative overestimation of stimulus size in the right position relative to the left position in right-handers, but no effect in left-handers. Although this finding was not quite robust, if there was any asymmetry at all for the visual size comparisons, it was in the direction opposite to that observed for the haptic size comparisons.

Although our participants were instructed to fixate the central fixation mark, we cannot claim with certainty that the circles presented to the left and right of the fixation mark were projected to the right and left cortical hemisphere, respectively. However, lateral asymmetries are not necessarily related to structural or functional differences between the cortical hemispheres. A prominent example is the relation of magnitude to the left-right dimension (cf. Walsh, 2003)^5^. Thus, whatever the sources of lateral asymmetries are, in our study handedness had different effects on size comparisons in the haptic and the visual modality. Hence, the underestimation of object size in the dominant hand, as compared to objects in the non-dominant hand, seems to be confined to the haptic modality and does not represent a universal, modality-independent phenomenon.

### Which factors drive the asymmetry of haptic size comparisons?

Having shown that strength differences between dominant and non-dominant limbs are not related to the observed bias in bimanual haptic size comparisons and that these biases are not an instance of modality-unspecific lateral asymmetries of size perception, the question remains which modality-specific factors underlie the bias. In the following we discuss three candidates: physical object weight, physical and perceived hand size, and size priors in bimanual actions.

In the present study – different from that of McPherson and Renfrew (1953) - the size differences between the hand-held discs were perfectly correlated with weight differences. In principle these physical weight differences could have contributed to the different lateral asymmetries found for left- and right-handers. However, this is quite unlikely for the following reasons. First, the weight differences may not have been noticed. Just-noticeable differences (JNDs), expressed as proportion of stimulus weight (Weber fraction), vary considerably (see, Jones, 1986, for review); they tend to be larger for bimanual than for unimanual comparisons (e.g., Saslow & Watkins, 1978) and to increase with smaller weights (i.e. < 50 g; e.g., Holway & Hurvich, 1937; Jones, 1986; Ross & Brodie, 1987). For the small weights of the present experiment, Weber fractions are typically larger than 0.20 (e.g., van Beek, King, Brown, & Di Luca, 2021; Holway & Hurvich, 1937). Thus, primarily the weight difference associated with the largest size difference of 3 mm could have been noted, for which the proportional weight difference was above 0.20 (for the smaller size differences the proportional weight differences were smaller).

Second, even if weight differences had been noted, such perceived differences cannot be crucial for the observed underestimation of object size in the dominant hand. Objects of same perceived size in both hands were physically larger and heavier in the dominant hand; had the stronger weight of the object in the dominant hand be perceived, this would have suggested a larger rather than a smaller object than in the non-dominant hand (Hirsiger et al., 2012). Thus, the physical differences in object weight that were correlated with object size in the present study should have counteracted the observed asymmetry in size comparisons rather than produced it, if they had any effect at all.

A second factor which could have contributed to the lateral asymmetry of haptic size comparisons is the shape or size of fingers and hands. Peters (1990) observed a larger width of thumb and index finger for the dominant than for the non-dominant hand, but not a larger length. The different widths of these fingers could influence size judgments with the finger-span method because wider fingers of the dominant hand would have to be opened more widely, possibly suggesting a larger object, than fingers of the non-dominant hand – which would contrast with the observed relative underestimation of size. Overall, however, the findings on physically different hand sizes are fairly mixed and give no obvious clue to a possible role for the asymmetries of perceived haptic size.

Although physical size differences between the left and right hand seem to be fairly variable, the dominant hand is rather consistently judged to be larger than the non-dominant hand, at least in right-handers (Linkenauger et al., 2009; Collier & Lawson, 2017). Again, this difference between hands is unlikely to be crucial because an experimentally increased perceived size of the hand results in an increased rather than reduced felt size of hand-held objects (Bruno & Bertamini, 2010).

The third possible contributor to the observed lateral bias of haptic size comparisons is the size prior, that is, the expected size of objects held in both hands. The role of expectations or priors for perception is well-established (e.g., de Lange et al., 2018). For bimanual size comparisons, specifically, such expectations could exist because of the functional specialization in bimanual tasks where the dominant and non-dominant hands typically operate on different spatio-temporal scales, micrometric-fast and macrometric-slow, respectively (cf. Ibbotson & Morton, 1981; Jordan et al., 2026; Peters, 1981; Guiard, 1987). Although we are not aware of independent evidence for such (relative) size expectations, there are at least two observations that are consistent with a role of them for the asymmetry of haptic size comparisons.

Whereas bimanual actions are statistically associated with different object sizes felt with both hands, smaller objects for the dominant than for the non-dominant hand, this is probably different for unimanual actions where the stronger dominant hand is likely preferred for larger objects, as posited by Wühr et al. (2024). Thus, the observed haptic bias of perceived size should be specific for *bimanual* size comparisons, that is, the comparison of the sizes of objects held in both hands. In contrast, when, for example, the sizes of objects held in the left or right hand are matched to the size of visual stimuli in an unimanual cross-modal task, one would not expect an asymmetry of haptic size perception or even an asymmetry opposite to that for the bimanual task. In fact, for haptic-visual size matching, which requires high-level judgments involving different frames of reference (cf. Héroux et al., 2024), absence of an asymmetry has been observed repeatedly (e.g., Collier & Lawson, 2017; pre-test results of Daneyko, Maravita, & Zanagno, 2020, and Frisco, Daneyko, Maravita, & Zanagno, 2023). An exception has been reported by Daneyko, Frisco, Maravita, & Zanagno (2025), who found smaller judgments for the right than for the left hand of right-handers. A possible source of this difference could have been a systematic order of tests, with the left hand preceding the right one. Notably, Daneyko et al. (2025, p.7) concluded that hand does not play a significant role in their specific cross-modal matching procedure.

The functional specialization of the hands in bimanual actions is pronounced when objects are acted upon, but not necessarily when they are only held. Both in the study of McPherson and Renfrew (1953) and in the present one, participants were allowed to roll the discs when held between two fingers of each hand. After repeated failures to replicate the findings of McPherson and Renfrew, Churchill (1971) hypothesized a crucial role of the fact that his stimuli (rods and discs) were fixed and could not be manipulated, although participants made small back-and-forth movements with thumb and forefinger on the objects.

### Perception is shaped by the requirements of actions

Although we are not aware of any specific theory that would predict the handedness-related bias in haptic size perception, the results of our study are consistent with the broad perspective of embodied perception. In particular they show that haptic perception in particular is shaped by the functions and capabilities of different body parts. To some extent this is a trivial fact: it is reflected, for example, in the variation of receptor densities across the surface of the human body, and in the distorted proportions of body parts in the so-called homunculus of the primary somato-sensory cortical areas. Obviously skilled movements of the fingers (in delicate manipulations) or of the tongue (in speaking) require higher sensory capabilities than movements of the proximal segments of arms or legs. However, in the supra-threshold range of perceptions the specificity of haptic perception for the action requirements of different body parts is not that trivial. The present findings are an example: they are unlikely to result from low-level sensory variations such as different receptor densities (or the associated sensory thresholds). At present their most likely origin, as it appears to us, are the size expectations for bimanual actions which contribute to inferences about the world given a certain sensory input.

Haptic perception differs from vision or audition by the direct physical contact between our body and objects in the environment. There is thus a time-honored claim that haptic perception marks reality (see Katz, 1925; Hacker, 1967). For example, no matter where we see an object, its ‘true’ location is the one where we can touch it or grasp it (or the ‘true’ location of a lamp-post is where we bump into it, no matter where we see it). The present findings show either that haptic perception does not mark reality, or that realities of the dominant and non-dominant hand are different. The latter statement appears to conflict with our conscious awareness of a unitary world where at each time each object has one place and one shape, but it is well in line with a variety of findings showing that object features such as their location, even locations of our hands, are represented differently (at different locations) in the human brain (e.g., Contier et al., 2024; Dijkerman & de Haan, 2007; Goodale, & Milner, 1992; Milner & Goodale, 2012; Rand & Heuer, 2018).

## Conclusions

In this study we replicated an old finding demonstrating that participants underestimate the size of an object in their dominant hand compared to the size of an object in their non-dominant hand. Moreover, we showed that this handedness-related bias in size comparisons seems confined to the haptic modality, and that it is not related to differences in strength between the dominant and the non-dominant hand. Identification of the specific differences in sensory inputs provided by the dominant and non-dominant hands and/or the size expectations that underlie the handedness-related bias in size judgments remains a challenge for the future; at present different priors for specific tasks find some indirect support. In more general terms, our findings show that haptic size perception is malleable by bodily dispositions, such as handedness, and therefore the impact of handedness on haptic size judgments represents an example for embodied perception in the haptic modality.

## Open Practices

The design and analysis of the experiment was pre-registered at OSF (https://osf.io/zc7fb). We report how we determined our sample size, all data exclusions (if any), all manipulations, and all measures in the study. The vocal responses in the haptic size-comparison task were noted on paper sheets by research assistants, summed up, and entered into Excel tables. The table containing the data from the haptic size-comparison task, and the raw data files from the visual size-comparison task have been published on the OSF platform (https://doi.org/10.17605/OSF.IO/XVE54). Programs for running the visual size-comparison task can be obtained from the first author upon request. Programs or code for fitting the psychometric functions can be obtained from the second author upon request.

## Data Availability

The table containing the data from the haptic size-comparison task and the raw data files from the visual size-comparison task have been published on the OSF platform ([https://doi.org/10.17605/OSF.IO/XVE54]).

## Footnotes

1 We used one-tailed tests for comparing PSEs from haptic size comparisons because we had directional hypotheses, based on the findings of McPherson and Renfrew (1953).

2 The Tukey criterion (Tukey, 1977) identifies a value as an outlier if it is 1.5 × IQR (IQR: inter-quartile range) smaller than the 25th percentile, or 1.5 × IQR larger than the 75th percentile, of the distribution. Our own experience suggests that the Tukey criterion often produces similar results as excluding cases that deviate more than 2.5 × SD from the mean.

3 We used a method proposed by Kerby (2014) for computing correlation coefficients as effect sizes.

4 We used two-tailed tests for comparing PSEs from visual size comparisons because we did not have directional hypotheses here, in contrast to the haptic size-comparison task.

5 Walsh (2003) proposed the existence of a generalized magnitude-processing system in the brain that uses a common metric to process space, time, and quantity. The fact that processing these dimensions shares cognitive and neural mechanisms should lead to interactions, which is supported by studies demonstrating interactions between number and space (e.g., Dehaene et al., 1993), for example.
